# Sea grapes powder with the addition of tempe rich in collagen: An anti-aging functional food

**DOI:** 10.12688/f1000research.55307.2

**Published:** 2022-04-19

**Authors:** Happy Kurnia Permatasari, Fahrul Nurkolis, Christopherous Diva Vivo, Sutamara Lasurdi Noor, Rahmawati Rahmawati, Son Radu, Hardinsyah Hardinsyah, Nurpudji Astuti Taslim, Nelly Mayulu, Defny Silvia Wewengkang, Mury Kuswari, Siti Chairiyah Batubara, William Ben Gunawan, Maizer Said Nahdi

**Affiliations:** 1 Department of Biochemistry and Biomolecular, Faculty of Medicine, Brawijaya University, Malang, 65145, Indonesia; 2 Department of Biological Sciences, Faculty of Sciences and Technology, State Islamic University of Sunan Kalijaga, Yogyakarta, 55281, Indonesia; 3 Dentistry, University of Indonesia, Depok, 16424, Indonesia; 4 Clinical and Public Health Nutrition Programme, University College London, London, WC1E 6BT, UK; 5 Food Technology Department, Sahid University of Jakarta, South Jakarta, 12870, Indonesia; 6 Food Sciences, Faculty of Human Ecology, Universiti Putra Malaysia, Serdang Selangor Darul Ehsan, 43400, Malaysia; 7 Applied Nutrition, Faculty of Human Ecology, IPB University, Bogor, 16680, Indonesia; 8 Clinical Nutrition, Faculty of Medicine, Hasanuddin University, Makassar, 90245, Indonesia; 9 Nutrition and Food, Faculty of Medicine, Sam Ratulangi University, Manado, 95115, Indonesia; 10 Pharmacy department, Faculty of Mathematics and Sciences, Sam Ratulangi University, Manado, 95115, Indonesia; 11 Nutrition Department, Faculty of health sciences, Universitas Esa Unggul, Jakarta, 11510, Indonesia; 12 Nutrition Sciences Department, Faculty of Medicine, Diponegoro University, Semarang, 50275, Indonesia

**Keywords:** Ageing, antioxidant, sea grapes, tempe, functional food

## Abstract

**Background:** This study aimed to determine the potential anti-aging effects of sea grapes and tempe (fermented soybeans) collagen particle size, by measuring the activities of anti-glycation, antioxidant, and tyrosinase inhibitors. **Methods**: Collagen was isolated from freeze-dried sea grapes and tempe powder and treated with different NaOH concentrations (0.10 M; 0.20 M; 0.30 M), and CH _3_COOH 1 M solution, separately. The collagen particle size was adjusted by stirring at 1000 rpm for 5 and 10 hours. 2,2-diphenyl-1-picrylhydrazyl (DPPH) was used to measure the antioxidant activity, and L-tyrosine and L-DOPA (l-3,4-dihydroxyphenylalanine) was used as a marker of tyrosine inhibition. **Results:**  The collagen treated with 0.10 M NaOH produced the highest collagen yield (11.65%), and the largest particle size (2455 nm). Additionally, this collagen, when treated for 5 hours, exhibited 24.70% antioxidant activity, 62.60% anti-glycation, 8.97% L-tyrosine, and 26.77% L-Dopa inhibition activities. Meanwhile, the collagen treated for 10 hours had a 9.98% antioxidant activity, 41.48% anti-glycation, 7.89% L-tyrosine, and 2.67% L-Dopa inhibition activity. **Conclusion:** Sea grapes and tempe collagen powder treated with 0.10 M NaOH and stirred for 5 hours, possess the best potential anti-aging properties as a functional food.

## Introduction

An unhealthy diet and excessive exposure to UV (ultra-violate) light can cause premature skin aging, leading to excesst melanin production (hyperpigmentation), and darker patches (depigmentation) ( Saeedi et al., 2019). Excessive UV light exposure can trigger oxidative stress, causing damage and apoptosis in skin cells. Oxidative stress occurs due to the increased intercellular levels of reactive oxygen species (ROS), which play an important role in the pathogenesis of aging and chronic disorders ( Peñalver et al., 2020; Park, 2013; Park et al., 2004). Consumption of high antioxidant functional foods in recent years has become popular as they can reduce oxidative stress damage. The presence of hydroxyl groups in antioxidant compounds acts as hydrogen donors to stabilize and prevent the formation of new ROS ( Pereira et al., 2009).

In some Asian countries, such as Malaysia, Indonesia, and the Philippines, sea grapes or *Caulerpa racemosa,* which are edible marine macroalgae, are believed to be functional foods or nutraceuticals packed with antioxidant properties that can delay or prevent premature skin aging ( Eren et al., 2019; Schumacker, 2015; Peñalver et al., 2020; Tanna et al., 2020; Yap et al., 2019; Pakki et al., 2020). Studies have explored several bioactive components in sea grapes, such as bioactive peptides, fibers (polysaccharides), polyphenols, flavonoids, antioxidants, and their distinctive compounds caulerpin ( Cao et al., 2021; Yang et al., 2015; Yap et al., 2019). In line with this, sea grapes extract tested in diabetic rats indicated a lowering effect on glucose levels, reduced aspartate aminotransferase, and alanine aminotransferase activities, and had a hepatoprotective effect ( Qudus et al., 2020).

Similar to sea grapes, tempe (fermented soyabeans) - a local Indonesian food - is known worldwide as a functional food, which also has a high antioxidant activity ( Kadar et al., 2020; Mani & Ming, 2017).

Premature aging can be exacerbated by an unhealthy diet as well. High glucose levels in the presence of limited insulin can trigger the glycation process, whereby glucose is attached to the proteins, lipids, and DNA of the skin, producing Advanced Glycation End-products (AGEs) ( Hantzidiamantis & Lappin, 2019; Kim et al., 2017). Consequently, AGEs can deactivate the antioxidants, attack collagen, and elastin, leaving the skin to lose moisture, become wrinkled, dull, and prone to damage and premature aging ( Gill et al., 2019). Consumption of antioxidants and collagen, such as those found in sea grapes and tempeh, can inhibit AGEs ( Aubry et al., 2020; Kadar et al., 2020; Yang et al., 2015).

Tyrosinase inhibition is another useful way of avoiding depigmentation. Tyrosinase transforms tyrosin to 3,4-dihydroxyphenylalanine (DOPA), then transforms DOPA to dopaquinone; which results in melanin at the end of the process ( Pillaiyar et al., 2017). As such this study aimed to determine the anti-aging potential effect of sea grapes and tempe collagen powder, by analyzing the activities of anti-glycation, antioxidant, and tyrosinase inhibitors.

## Methods

### Sample preparation

Sea grapes ( *Caulerpa racemosa*) were rinsed and cleaned with the use of CO _2_ free water. The soybean-based tempe is mixed with sea grapes (0.25:1) with a blender, and frozen at −22°C for 12 hours. Samples were dried using a freeze-dryer (Lyovapor ^TM^ L-200) for 24 hours, which resulted in 0.3-0.5 mm powder.

### Water and ash content determination

The determination of water content was based on the Association of Official Analytical Chemists (AOAC) drying method (Thermogravimetry) ( Latimer, 2019) ( Table 1), and the content was calculated by using the following formula:

$$\text{Water content}\;\left(\%\right)=\frac{W1-W2}{W1-W0}\times 100$$

W0 = Weight of empty cup

W1 = Weight of the cup + initial sample (before heating in the oven)

W2 = Weight of cup + initial sample (after cooling in a desiccator)

The procedure for determining the ash content was also with the use of the AOAC method ( Latimer, 2019), and the content was calculated by using the following formula:

$$\mathrm{Ash}\;\text{content}\;\left(\%\right)=\frac{\text{Weight of the bowl after heated}-\text{Constant weight of empty bowl}}{\text{Sample weight}}\times 100$$

**Table 1.** Water and ash content.

Ash (%)	Water(%)
2.65 ± 0.50	3.42 ± 1.05

### Collagen isolation

Collagen from sea grapes and tempe powder is isolated by treating the samples (ready-to-eat dry products) with three variations of NaOH concentrations (0.10 M; 0.20 M; 0.30 M) with a ratio of 1:10 (w/v), for 48 hours. The samples were then dried with the use of a freeze dryer (Lyovapor ™ L-200) and treated with 1 M CH _3_COOH solution at a ratio of 1:10 (w/v), for 24 hours. Whatman filter paper (Grade 1) was used to obtain the filtrate. Lastly, the collagen obtained was once again dried with a freeze dryer.

### Collagen size-reduction

The optimal NaOH treated collagen is dissolved with distilled water (1:2 (v/v)) and spun for 5 and 10 hours with a magnetic stirrer (1000 rpm) to establish size transformation. The size of the particles was measured by using the Particle Size Analyzer (PSA), and the antioxidant activity was tested with DPPH Assay (2,2-diphenyl-1-picrylhydrazyl), antiglycation, and tyrosinase inhibitors.

### Antioxidant activity measurement

The enzyme-linked immunosorbent assay (ELISA, Sigma #CS0790) was used to determine the antioxidant activity of DPPH ( Batubara et al., 2015). 100 μL of each sample along with 100 μL of DPPH (0.3 mM) was added to the 96-well microplate and incubated for 30 minutes in a dark room. The absorbance was measured by using an ELISA reader at a wavelength of 517 nm ( Underlying data) ( Nurkolis, 2021). The antioxidant activity is calculated as follows:

$$\text{Inhibition}\;\left(\%\right)=\frac{\left(A0-A1\right)}{A0}\times 100\%$$

A0 = Absorbance of blank

A1 = Absorbance of standard or sample

### Anti-glycation activity measurement

The anti-glycation measurement ( Table 2) was carried out as previously described ( Povichit et al., 2010) ( Underlying data) ( Nurkolis, 2021). All the test solutions were incubated at 60°C for 40 hours. After incubation, the aliquots (100 μL) were pipette into a 96-well plate. The relative amount of glycated Bovine Serum Albumin (BSA) was measured using a fluorometer at an excitation wavelength of 370 nm, and emission of 440 nm.

**Table 2.** The composition of the solution in the anti-glycation activity test.

Materials	Solution A (Glycation control) (μL)	Solution B (Control corrector) (μL)	Solution C (Sample) (μL)	Solution D (Sample corrector) (μL)
Phosphate buffer 200 mM pH 7.4 (KH _2_PO _4_ 0.2 M + K _2_HPO _4_ 0.2 M in distilled water)	200	200	200	200
BSA 20 mg/mL	80	80	80	80
Glucose 235 mM	40	-	40	-
Fructose 235 mM	40	-	40	-
Extract/Aminoguanidin	-	-	80	80

### Tyrosinase inhibitory activity measurements

The tyrosinase enzyme inhibitory activity was measured as previously described ( Batubara et al., 2015). L-tyrosine and L-DOPA (l-3,4-dihydroxyphenylalanine) were used as substrates (MyBioSource #MBS9301852), and kojic acid as positive controls ( Table 5) ( Underlying data) ( Nurkolis, 2021). Samples were dissolved with dimethyl sulfoxide (DMSO) as a stock solution. The concentration variant was prepared by dissolving collagen with a phosphate buffer (pH of 6.5). A total of 70 μL of solution along with 30 μL of tyrosinase enzyme (Sigma, 333 units mL ^-1^ in phosphate buffer solution was added) was pipetted into the 96-well plate, and the mixture was incubated for 5 minutes. To this mixture, 110 μL of the substrate (L-tyrosine 2 mM) was added and incubated at 37°C for 30 minutes. The absorbance was measured at a wavelength of 492 nm, by using the microplate reader (Spectrophotometer).

## Data analysis

Statistical analyses were performed by using SPPS 26.0 for the Windows version. The differences between samples are analyzed based on the antioxidant activity, anti-glycation activity, and tyrosinase inhibition activity tests. The data obtained from three replications (triples) were analyzed by ANOVA at 95% CI (p < 0.05). The result is defined as significant if the p-value is < 0.05.

## Results

### Ash and water contents

Table 1 shows the triplicate process resulted in 3.42 (± 1.05%) water content and 2.65 (± 0.50%) ash content.

### Collagen yield

The collagen yield obtained by each concentration is shown in Table 3. The isolation with NaOH 0.10 M produced the highest collagen yield (p < 0.05), this showed that there was a significant difference in the yield of the three variations of NaOH and CH3COOH treatment. Levenes’ test of homogeneity of variants was p = 0.397 (p > 0.05).

**Table 3.** The yield of isolated collagen with NaOH and CH _3_COOH variations

NaOH+CH _3_COOH concentrations (M)	Collagen yields (%)
0.10	11.65
0.20	8.70
0.30	4.98

### Collagen particle size

Particle Size Analyzer (PSA) was used to determine the collagen particles’ size. The collagen yields ranged from 1012 nm to 2455 nm, with the highest DPPH and glycation inhibitions at 2455 nm (11.74% and 62.76%, respectively) ( Table 4). In addition to producing significantly different yields, different treatments across the three samples were also significantly different in particles size (p = 0.000), with p > 0.05 homogeneity. The collagen with the largest particle size of 2455 nm was obtained from 0.10 M NaOH treatment for 5 hours ( Table 4).

**Table 4.** Particle size, antioxidant activity and glycation inhibition

Collagen treatment	Particle size (nm)	DPPH inhibition (%)	Glycation inhibition (%)
NaOH 0.10 M	2455	11.74 ^*^	62.76 ^*^
NaOH 0.20 M	1012	8.13 ^*^	42.50 ^*^
NaOH 0.30 M	1922	12.39 ^*^	57.43 ^*^
NaOH 0.10 M (5 Hours)	1482	24.70 ^*^	62.60 ^*^
NaOH 0.10 M (10 Hours)	1568	9.98 ^*^	41.48 ^*^

* Shows significant difference at p = 0.05.

### Antioxidant, anti-glycation, and tyrosinase inhibitor activity

The 0.10 M NaOH treatment for 5 hours, resulted in 24.70% and 62.60% antioxidant and anti-glycation activities, respectively ( Table 4). However, treatment with 0.10 M NaOH for 10 hours resulted in 9.98% antioxidant and 41.48% anti-glycation activities. Additionally, treatment with 0.10 M NaOH for 5 hours inhibited 8.97% of L-tyrosine and 26.77% of L-Dopa activities ( Table 5).

**Table 5.** Anti-tyrosinase activity of collagen at 1000 mg/L.

Collagen	Tyrosinase inhibition by substrate (%)	
	*L-Tirosina*	*L-Dopa*
NaOH 0.10 M (5 Hours)	8.97	26.77 ^*^
NaOH 0.10 M (10 Hours)	7.89	2.67 ^*^

Kojic Acid ^IC^50: 8.90 mg/L.

* Shows significant difference at p = 0.05.

## Discussion

Based on the ash and water content analysis, the powder made from sea grapes and tempe is considered safe to consume, based on the Indonesia National Standard (SNI) No. 01-4320-1996 regulations for food in powder form or powder extract (3% maximum water content). Moreover, pre-treatment was done to remove the non-collagen proteins, as well as assess the number of pure collagen proteins in the final product. Collagen is usually insoluble in alkaline solutions, however, NaOH treatment is commonly used in the collagen extraction process as it can significantly minimize collagen loss, compared to other alkaline solutions ( Liu et al., 2015). In this study collagen from sea grapes and tempe powder treated with 0.10 M NaOH produced the highest yield, which showed the effectiveness of the extraction process. As indicated by Potaros and colleagues, the difference in yield can be caused by the extraction method, such as the concentration of a solution in the non-collagen protein separation process, and the type of material used ( Potaros et al., 2009). Therefore, treatment with variations of NaOH concentration could affect the collagen yields (%), particle size, DPPH inhibition (%), and anti-glycation produced (%).

The collagen particle measurements in this study ranged from 1012 to 2455 nm ( Table 4), which was too large to be considered nanoparticles (10-1000 nm) ( Mohanraj & Chen, 2007). It is important to control the size of nanoparticles since different sizes may result in adverse effects such as cytotoxic ( Perde-Schrepler et al., 2019). Therefore, further optimization was carried out to reduce the collagen particle size of the 0.10 M NaOH treatment, through stirring for 5 or 10 hours. It is necessary to reduce the particle size to increase its absorption by the digestive system ( Mohanraj & Chen, 2007) and bypass the transport barriers in biological tissues ( Islam, Barua, & Barua, 2017). In a study by Mohanrja et al., reducing the particle size should be through the hydrolysis process, and not by a mechanical process such as stirring, as it can re-solidify or coagulate the collagen ( Mohanraj & Chen, 2007). However, the hydrolysis process was avoided in this study, as it might have broken down other important compounds, such as antioxidants. Mechanical stirring for 5 hours, resulted in an almost 2-fold reduction in the size of the collagen particles. However, stirring for 10 hours did not reduce the particle size due to the reasons described by Mohanraj and Chen (2007).

The treatment with 0.10 M NaOH ( Table 4), produced the largest particle size with the highest anti-glycation activity compared to other concentrations, however, its antioxidant activity was lower compared to 0.30 M NaOH. The percentage of antioxidants produced is similar to commercial collagen ( ^IC^50), which is greater than the result in the study by Fauzi (2018). At 0.10 M NaOH treatment for 5 hours had better anti-glycation activity than at 10 hours. The resulting anti-glycation activity was higher when compared to the 17.74% activity of the collagen produced in Fauzi’s dissertation research ( Fauzi, 2018).

**Figure 1.**
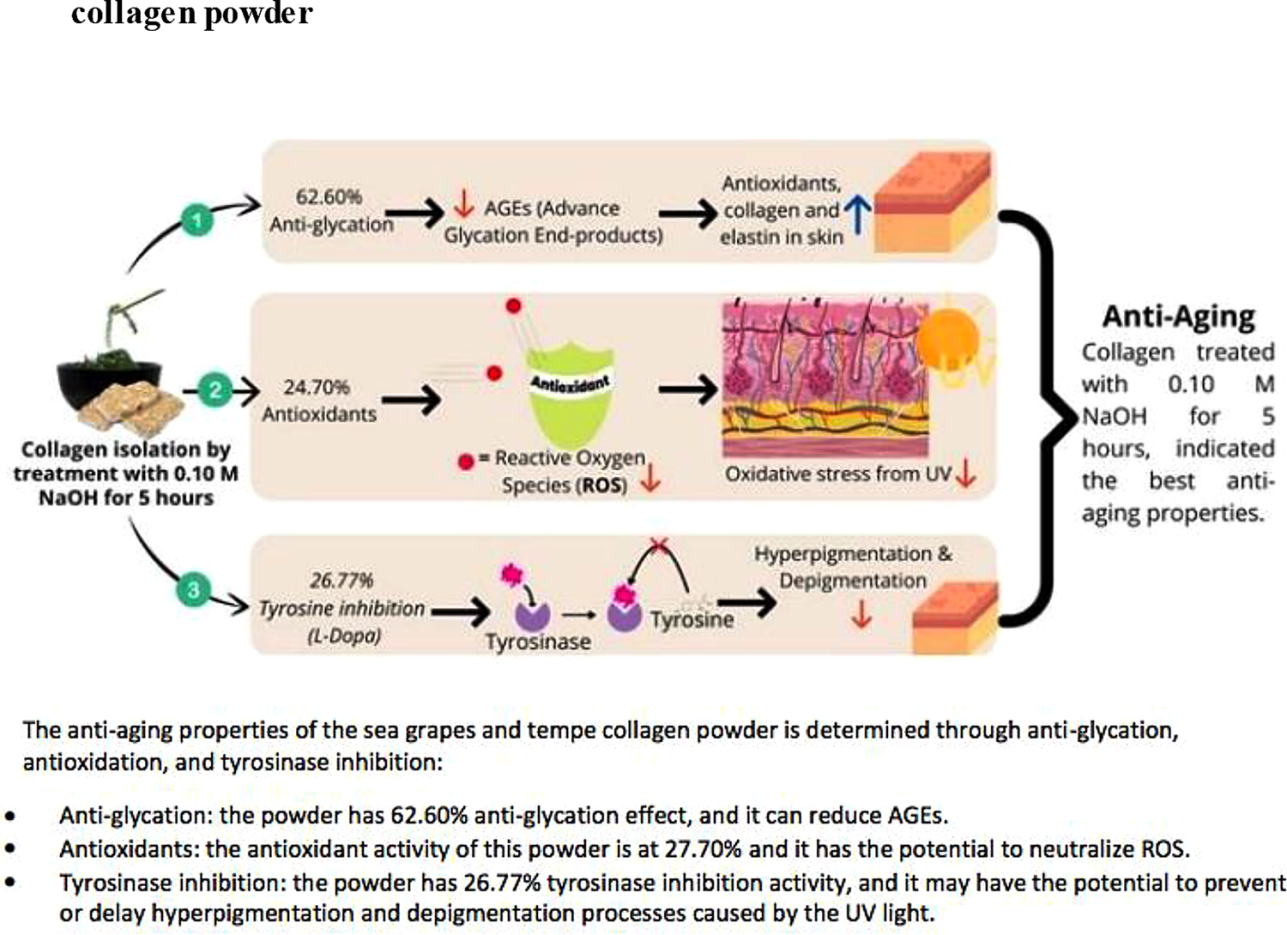
Anti-glycation, antioxidant, and tyrosinase inhibition of sea grapes and tempe collagen powder.

Excessive melanin production or hyperpigmentation caused by exposure to excessive UV rays can lead to dark skin or depigmentation ( Saeedi et al., 2019). Tyrosine inhibition can reduce excessive melanin production, which can prevent skin damage. The results of this study showed that treating L-tyrosine and L-DOPA substrates for 5 hours had a greater tyrosinase enzyme inhibitory activity, compared with treatment for a longer period ( Table 5) ( Figure 1). In the Fauzi study, commercial collagen did not show tyrosinase enzyme inhibitory activity at 1000 mg/L and exhibited lower activity than the collagen obtained in the present study ( Fauzi, 2018).

Sea grapes and tempe powder combined with a variety of food additives can be used by manufacturing companies as functional foods or anti-aging nutraceuticals, by NaOH (0.10 M) and CH _3_COOH (1 M) treatment at 1000 rpm for 5 hours ( Figure 1). However, this *in-vitro* pilot study has the potential to be a basic reference for pre-clinical research. Further trials are needed to determine the continued efficacy of this study.

## Conclusion

Sea grapes and tempe collagen powder as functional foods or nutraceuticals exhibit potential anti-aging properties. Based on the anti-glycation, anti-tyrosinase, and antioxidant activities, the collagen of this powder treated with 0.10 M NaOH for 5 hours, has the most optimal anti-aging effect. Manufacturers seeking to produce anti-aging food products rich in collagen can use this method for determining the optimal powder formulation, however extensive trials are still needed to further analyze its clinical effects.

## Data availability

### Underlying data

Figshare: Sea grapes powder with addition of tempe rich in collagen: An anti-aging functional food.

DOI: https://doi.org/10.6084/m9.figshare.15072597.v3 ( Nurkolis, 2021).

The project contains the following underlying data:

• Raw data: Water and ash content, antioxidant activity, glycation inhibition activity, particle size, and anti-tyrosinase activity of the collagen. The chemical composition of the solution in the anti-glycation activity test.

Data are available under the terms of the Creative Commons Zero “No rights reserved” data waiver ( CC0 1.0 Public domain dedication).

## Author contributions

All authors contributed to the writing and revision of this article, and all authors have read and approved the final manuscript. H. K. P. and F. N. gathered study ideas, designed the experiments, analyzed data, and compiled manuscripts. N. A. T., H. H., N. S., M. K., S. R., R. R., and N. M. analyzed and interpreted data and critically revised the manuscript. The F. N., S. L. N., D. S. W., and H. K. P. conducted experiments, analyzed biochemistry, and critically revised the manuscript. N. M., S. C. B., W. B. G., and C.D.V., implemented experimental protocols, assisted in statistical analysis, interpreted data, and critically revised manuscripts. All writers read and approve the final manuscript.
