# Supplementary material for: Sea grapes powder with the addition of tempe rich in collagen: An anti-aging functional food
Source: F1000Res. 2022 Jul 27;10:789. Originally published 2021 Aug 11. [Version 3] doi: 10.12688/f1000research.55307.3 (PMC9471220; doi:10.12688/f1000research.55307.3)
Supplement: Peer review history

# Peer review: Reviewer response for version 3

**DOI:** 10.5256/f1000research.136618.r145556

**Published:** 2022-09-13

**Authors:** Manoj Kumar, Vijay Sheri

**Affiliations:** 1 Chemical and Biochemical Processing Division, ICAR-Central Institute for Research on Cotton Technology, Mumbai, India; 2 Department of Biology, East Carolina University, Greenville, USA

We agree with the revisions performed by the authors. Manuscript may be accepted in current format.

Is the work clearly and accurately presented and does it cite the current literature?

Yes

If applicable, is the statistical analysis and its interpretation appropriate?

Yes

Are all the source data underlying the results available to ensure full reproducibility?

Yes

Is the study design appropriate and is the work technically sound?

Yes

Are the conclusions drawn adequately supported by the results?

Yes

Are sufficient details of methods and analysis provided to allow replication by others?

Yes

Reviewer Expertise:

Food Biochemistry

We confirm that we have read this submission and believe that we have an appropriate level of expertise to confirm that it is of an acceptable scientific standard.

# Peer review: Reviewer response for version 3

**DOI:** 10.5256/f1000research.136618.r145557

**Published:** 2022-09-06

**Authors:** Yasuyuki Irie

**Affiliations:** 1 Department of Nutritional Science, Faculty of Health and Welfare Science, Okayama Prefectural University, Okayama, Japan

The revised version of the article should be approved. The aims and research methods are adequate; results are presented accurately; and the conclusions are justified and supported by the data or supporting material.

Is the work clearly and accurately presented and does it cite the current literature?

Partly

If applicable, is the statistical analysis and its interpretation appropriate?

Yes

Are all the source data underlying the results available to ensure full reproducibility?

Yes

Is the study design appropriate and is the work technically sound?

Yes

Are the conclusions drawn adequately supported by the results?

Partly

Are sufficient details of methods and analysis provided to allow replication by others?

Yes

Reviewer Expertise:

Pharmacology and Nutritional Science

I confirm that I have read this submission and believe that I have an appropriate level of expertise to confirm that it is of an acceptable scientific standard.

# Peer review: Reviewer response for version 2

**DOI:** 10.5256/f1000research.125028.r135865

**Published:** 2022-07-18

**Authors:** Manoj Kumar, Vijay Sheri

**Affiliations:** 1 Chemical and Biochemical Processing Division, ICAR-Central Institute for Research on Cotton Technology, Mumbai, India; 2 Department of Biology, East Carolina University, Greenville, USA

**Update 13 September 2022: The details of the co-reviewer were added post the publication of this report and the language used was updated to reflect this (i.e., 'I' was edited to 'We').**

 We have carefully evaluated manuscript entitled “Sea grapes powder with the addition of tempe rich in collagen: An anti-aging functional food”. The manuscript can be followed well, but there are some minor concerns. Moreover, English language also need minor revisions. Punctuations also need to be checked carefully throughout the manuscript.

**Material and Methods:**

 Authors must add a flow diagram showing methodology followed in the experimentation and also show the analysis performed at each stage. This flow diagram will definitely improve the readability of the manuscript.

**Result and Discussion:**

 Authors have presented their findings in a well manner with appropriate discussions, but we are not satisfied with recent works carried out on similar area. Most of the cited articles in the discussion section are not sufficient. It is suggested to improve the manuscript considering this specific aspect. We wish to see a revised version of the manuscript with a good discussion throughout the result and discussion section with recent literature.

Is the work clearly and accurately presented and does it cite the current literature?

Yes

If applicable, is the statistical analysis and its interpretation appropriate?

Yes

Are all the source data underlying the results available to ensure full reproducibility?

Yes

Is the study design appropriate and is the work technically sound?

Yes

Are the conclusions drawn adequately supported by the results?

Yes

Are sufficient details of methods and analysis provided to allow replication by others?

Yes

Reviewer Expertise:

Food Biochemistry

We confirm that we have read this submission and believe that we have an appropriate level of expertise to confirm that it is of an acceptable scientific standard, however we have significant reservations, as outlined above.

# Response

**Published:** 2022-07-27

**Authors:** Fahrul Nurkolis

**Affiliations:** State Islamic University of Sunan Kalijaga (UIN Sunan Kalijaga Yogyakarta), Indonesia

Dear *2nd Reviewer*,

*Manoj Kumar, PhD*

 Chemical and Biochemical Processing Division, ICAR-Central Institute for Research on Cotton Technology, Mumbai, India.

 We greatly appreciate all of your helpful comments and suggestions in our article. We have considered those comments carefully and revised the manuscript based on those suggestions.

 Now, the latest version of our article has been published (Version 3). We hope that you will return to review this article. 

 The authors made minor improvements in accordance with the suggestions by reviewers 1 and 2 (Dr. Yasuyuki Irie and Dr. Manoj Kumar), the following improvements:

 1. Added "Figure 1. Graphical Methodology of Experimental Study" in the method section.

 2. Addition of several sentences and references in the discussion section, such as references: Kageyama *et al*., 2021; Egorikhina *et al*., 2021; and Kumar *et al*., 2022.

 3. Improved English language and punctuations in main/whole articles.

 Thank you!

# Peer review: Reviewer response for version 2

**DOI:** 10.5256/f1000research.125028.r135111

**Published:** 2022-05-09

**Authors:** Yasuyuki Irie

**Affiliations:** 1 Department of Nutritional Science, Faculty of Health and Welfare Science, Okayama Prefectural University, Okayama, Japan

I think that the revised version corrected the parts identified in the peer review and made the content suitable for indexing.

- Is the work clearly and accurately presented and does it cite the current literature?
  Yes
- Is the study design appropriate and is the work technically sound?
  Yes
- Are sufficient details of methods and analysis provided to allow replication by others?
  Yes
- If applicable, is the statistical analysis and its interpretation appropriate?
  Yes
- Are all the source data underlying the results available to ensure full reproducibility?
  Yes
- Are the conclusions drawn adequately supported by the results?
  Yes

Is the work clearly and accurately presented and does it cite the current literature?

Partly

If applicable, is the statistical analysis and its interpretation appropriate?

Yes

Are all the source data underlying the results available to ensure full reproducibility?

Yes

Is the study design appropriate and is the work technically sound?

Yes

Are the conclusions drawn adequately supported by the results?

Partly

Are sufficient details of methods and analysis provided to allow replication by others?

Yes

Reviewer Expertise:

Pharmacology and Nutritional Science

I confirm that I have read this submission and believe that I have an appropriate level of expertise to confirm that it is of an acceptable scientific standard.

# Response

**Published:** 2022-07-27

**Authors:** Fahrul Nurkolis

**Affiliations:** State Islamic University of Sunan Kalijaga (UIN Sunan Kalijaga Yogyakarta), Indonesia

Dear *1st Reviewer*,

*Professor Yasuyuki Irie, MD., PhD*

 Department of Nutritional Science, Faculty of Health and Welfare Science, Okayama Prefectural University, Okayama, Japan.

 We greatly appreciate all of your helpful comments and suggestions in our article. We have considered those comments carefully and revised the manuscript based on those suggestions.

 Now, the latest version of our article has been published (Version 3). We hope that you will return to review this article.

 Thank you!

# Peer review: Reviewer response for version 1

**DOI:** 10.5256/f1000research.58871.r119984

**Published:** 2022-01-31

**Authors:** Yasuyuki Irie

**Affiliations:** 1 Department of Nutritional Science, Faculty of Health and Welfare Science, Okayama Prefectural University, Okayama, Japan

The authors aimed to determine the anti-aging potential effect of sea grapes and tempe collagen powder, by analyzing the activities of anti-glycation, antioxidant and tyrosinase inhibition. In general, the article clearly and accurately presented the data and their interpretations citing the current literature.

 As a *in vitro* pilot study, the study design is appropriate and the study has academic merit. Although I am not a specialist for food processing, the methods seem to be appropriate and can be replicated by other researchers. All the source data underlying the results available to ensure full reproducibility. The conclusions were drawn adequately and supported by the results.

 One of the new findings they presented in this study is the development of a method to optimize the collagen particle size. Therefore, it is necessary to fully explain the significance of optimizing collagen particle size and the previous studies. That is the comment for the inquiry "Is the work clearly and accurately presented and does it cite the current literature?"

 In the last part of the abstract, the authors assert that "Sea grapes and tempe collagen powder treated with 0.10M NaOH and stirred for 5 hours, as functional foods have anti-aging properties." However, as stated in the last part of the Discussion in the text, this study was an " *in vitro* pilot study," and it cannot be concluded that functional foods have anti-aging properties. Therefore, the wording of the abstract should be changed appropriately. That is the comment for the inquiry "Are the conclusions drawn adequately supported by the results?"

Is the work clearly and accurately presented and does it cite the current literature?

Partly

If applicable, is the statistical analysis and its interpretation appropriate?

Yes

Are all the source data underlying the results available to ensure full reproducibility?

Yes

Is the study design appropriate and is the work technically sound?

Yes

Are the conclusions drawn adequately supported by the results?

Partly

Are sufficient details of methods and analysis provided to allow replication by others?

Yes

Reviewer Expertise:

Pharmacology and Nutritional Science

I confirm that I have read this submission and believe that I have an appropriate level of expertise to confirm that it is of an acceptable scientific standard, however I have significant reservations, as outlined above.
